# The Weight of HLA-DPA1 rs3077 Single Nucleotide Polymorphism in Prostate Cancer, a Multicenter Study

**DOI:** 10.1155/2021/5539851

**Published:** 2021-04-21

**Authors:** Mohammed Jayed Alenzi, Amany A. Ghazy, Diaa-Eldin Taha

**Affiliations:** ^1^Department of Surgery, College of Medicine, Jouf University, Sakaka, Saudi Arabia; ^2^Department of Pathology (Microbiology and Immunology Unit), College of Medicine, Jouf University, Sakaka, Saudi Arabia; ^3^Departments of Microbiology and Medical Immunology, Faculty of Medicine, Kafrelsheikh University, Kafrelsheikh, Egypt; ^4^Department of Urology, Faculty of Medicine, Kafrelsheikh University, Kafrelsheikh, Egypt

## Abstract

Prostate cancer (PCa) has almost the highest genetic transmission that mimics an autosomal dominance hereditary pattern of cancers in some families. Its incidence in Arab countries was reported to be steadily increasing. *Aim*. To determine the relevance of HLA-DPA1 rs3077 (A/G) SNP with prostate cancer's risk and/or severity. *Subjects and Methods*. Forty PCa patients and forty age matched patients with benign prostatic hyperplasia (BPH), as a control group, were enrolled in the study. Serum levels of urea, creatinine, total prostate-specific antigen (PSA), and free PSA were measured. PSA ratio was determined as well. Genotyping of HLA-DPA1 rs3077 (A/G) SNP was done using real-time PCR. *Results*. The measured lab parameters, except free PSA, were significantly higher among PCa patients in comparison to controls (*P* < 0.001^*∗*^). Moreover, PSA ratio was significantly high among PCa patients (*P* < 0.001^*∗*^). HLA-DPA1 rs3077 GG genotype was more frequent in PCa patients and the associated OR was 2.546 (*P*=0.059), while AA genotype was more frequent in the control group and the associated OR was 0.145 (*P*=0.081). Frequency of G allele was higher among PCa patients than the control group while A allele frequency was significantly decreased (*P*=0.034^*∗*^) (protective allele). On multivariate analysis, there is no significant correlation found between HLA-DPA1 rs3077 SNP and PSA ratio (OR = 4.5, 95% CI = 1.2–17.4, *P*=0.856). *Conclusion*. HLA-DPA1 rs3077 G allele could be a risk factor for prostate cancer. However, HLA-DPA1 rs3077 SNP has no relation to PCa severity.

## 1. Background

Prostate cancer (PCa) is considered one of the most common tumors among adult males [1]. It is usually diagnosed at late stage after metastasis and emergence of therapeutically resistant tumor cells which in turn confers a high mortality rate [2].

Pathogenesis of PCa is multifactorial including genetic, hormonal, and environmental factors [3]. In this context, genome-wide association studies (GWAS) have identified several PCa susceptibility genes within region 7p21.3 and 18p11.22. These gene polymorphisms can explain part of the genetic etiology of PCa [1].

Human leukocyte antigen (HLA) alleles play a crucial role in activation of immune cells that are responsible for clearance of tumor cells. It may be an important genetic host attribute [4, 5]. The great variability in HLA genes has encouraged researchers to investigate the role of HLA genetic variations in many diseases [4–6]. We have performed studies on the association between HLA SNP and different diseases such as breast cancer (BC), hepatitis B virus (HBV), and hepatitis C virus (HCV) [6–8]. Results were controversial as we found significant association between HLA-DP1 rs3077 AA genotype and BC risk (OR = 4.5, 95% CI = 1.2–17.4, *P* < 0.05^*∗*^), and no association was found between HLA-DQ rs3920 and BC [6]. However, the relevance of HLA SNP and PCa is still unclear. Thus, the present study aimed to determine the significance of HLA-DPA1 rs3077 (A/G) SNP with the risk and/or severity of prostatic cancer.

## 2. Participants and Methods

### 2.1. Study Design

This case-control multicenter study enrolled 80 participants attending Urology Department, Prince Mutaab Bin AbdelAziz Hospital, Sakakah, and Kafrelsheikh University Hospital. Participants were divided into 2 groups: group 1 included 40 patients with pathologically confirmed malignant PCa and group 2 included 40 patients with benign prostatic hyperplasia (BPH) as a control group. Exclusion criteria include secondary PCa, hepatic decompensating, and renal failure.

### 2.2. Sample Collection

Five ml blood samples were collected in plain tubes (3 ml for serum separation) and EDTA tubes (2 ml for DNA extraction).

### 2.3. Clinical Evaluation

Enrolled patients were assessed clinically by history, examination including digital rectal examination (DRE), and international prostate scoring system (IPSS). Patients who have PSA higher than 4 ng/ml were subjected to trans-rectal ultrasound (TRUS) and biopsy using standard 6 cores. Moreover, TRUS helps to attain prostate volume. Grading of pathologic results was elaborated utilizing Gleason grading score [9].

### 2.4. Biochemical Investigations

Serum levels of urea, creatinine, total prostate-specific antigen (PSA), and free PSA were measured for all participants. PSA ratio (free-to-total PSA) was determined too.

### 2.5. Genotyping of HLA-DPA1 rs3077 (A/G) SNP

DNA was extracted from blood samples in EDTA tubes using QIAamp-spin columns (QIAamp DNA Blood Mini Kit, Applied Biosystems, Life Technologies, California, USA) according to the manufacturer instructions. After assessing DNA purity and concentration using nanodrop spectrometer, samples were subjected to PCR discrimination of HLA-DPA1 rs3077 (A/G) genotypes, using real-time PCR (StepOne® Real-Time PCR, Applied Biosystem, Life Technologies, Carlsbad, California, USA). In PCR tubes, 2 *μ*l genomic DNA, 1.25 *μ*l TaqMan SNP Genotyping Assay, 12.5 *μ*l TaqMan PCR master mix, and 9.25 *μ*l DNase-free water were mixed [8]. The reaction volume was 25 *μ*l and the thermal cycler conditions were adjusted at 95°C for 10 min, 40 cycles of 92°C for 15 sec, 60°C for 1 min, and 72°C for 30 sec and then a final extension at 72°C for 7 min. Results analysis was depending on that increase in VIC dye means homozygosity for the wild alleles (HLA-DPA1 rs3077 AA), increase in FAM dye indicates homozygosity for the mutant alleles (HLA-DPA1 rs3077 GG), and if both fluorescence signals increase, this indicates heterozygosity (HLA-DPA1 rs3077 AG) [8].

### 2.6. Statistical Analysis of the Data

Data were fed to the computer and analyzed using IBM SPSS software package version 20.0 (Armonk, NY: IBM Corp). Kolmogorov-Smirnov normality test was used to examine if variables are normally distributed. Comparisons between groups for categorical variables were assessed using chi-square test. Student's *t*-test was used to compare two groups for normally distributed quantitative variables, Mann–Whitney test was used for abnormally distributed quantitative variables; to compare between two studied groups, odds ratio (OR) was used to calculate the ratio of the odds and 95% confidence interval of an event occurring in one risk group to the odds of it occurring in the nonrisk group, Hardy-Weinberg. The population of the studied sample was explored to find its equilibrium with Hardy-Weinberg equation. Significance of the obtained results was judged at the 5% level.

## 3. Results

### 3.1. Subjects Demographic and Clinical Data

Subjects' demographic and clinical data are illustrated in Table 1. There was not any statistically significant difference between the studied groups regarding age (*P*=0.106). However, the prostate volumes were markedly increased among PCa patients in comparison with benign prostatic hyperplasia control group (*P*=0.006^*∗*^).

Medical history of PCa patients revealed that 82.5% of patients did not suffer from any chronic disease, 7.5% were hypertensive, 2.5% have diabetes mellitus, 2.5% have hepatic fibrosis, and 5% have nephropathy. While for the control group, 82.5% were normal, 10% were hypertensive, and 7.5% have nephropathy. There was not any statistical difference between the studied groups (*P*=0.673).

Surgical history of PCa patients revealed that 87.5% of patients were normal, 10% had surgery to remove urinary stone, and 2.5% had open heart surgery. While for the control group, 95% were normal and 5% had stone surgery. There was not any statistical difference between the studied groups (*P*=0.409).

### 3.2. Biochemical Investigations

The mean serum levels of urea, creatinine, total PSA, and PSA ratio (free-to-total PSA) were markedly increased among PCa patients in comparison to the control group (*P* < 0.001^*∗*^) (Table 1). Gleason grading of PCa patients clarified that 35.1% were grade I, 8.1% were grade II, 13.5% were grade III, 32.4% were grade IV, and 10.8% were grade V.

### 3.3. HLA-DPA1 rs3077 (A/G) SNP

HWE was done to check for any possible deviations within all participants regarding the studied genotype frequencies and no deviation was observed (*P* > 0.05).

Allelic discrimination of HLA-DPA1 rs3077 SNP clarified that the frequency of GG genotype was higher among PCa patients in comparison with the control group (42.5% vs. 22.5%; *P*=0.056) and the odds ratio (OR; 95% CI) was 2.546 (0.964–6.726). However, the frequency of AA genotype was significantly higher among the control group in comparison with PCa patients (15% vs. 2.5%; *P*=0.048^*∗*^) and the odds ratio (OR; 95% CI) was 0.145 (0.017–1.268). Comparison based on allele showed significantly higher frequency of G allele among PCa patients than the control group (70% vs. 53.8%; *P*=0.034^*∗*^) and higher frequency of A allele among the control group in comparison with PCa patients (46.3% vs. 30%; *P*=0.034^*∗*^). Thus, there was a statistically significant increase in HLA-DPA1 rs3077 G allele among PCa patients and A allele among the controls (*P*=0.034^*∗*^ for both) (Table 2).

Correlation analysis between PSA ratio and different genotypes in the PCa group did not show any significant association (*P*=0.856) (Table 3).

## 4. Discussion

Prostate cancer was stated to have the highest degree of genetic transmission with suggestion to mimic an autosomal dominance hereditary pattern in some families [10].

In the Arab World, the PCa incidence rate ranges between 5.5% in Saudi Arabia and 39.2% in Lebanon [11]. However, the incidence and mortality rates were reported to be steadily increasing due to poor knowledge and attitude towards PCa examination and screening practices.

GWAS was conducted on four populations to understand the contribution of genetic variants in PCa penetrance [1]. They reported that some SNP-SNP interactions are involved in cancer progression either by regulating the expression of their genes or the nearby genes' proteins. Thus, presence of multiple risk alleles is more predictive than a single allele. Another study has reported that SNPs confer a cumulative risk of PCa development [12].

In addition, it is known that tumors emerge when the immune surveillance fails and HLA system plays a major role in this surveillance by activating T-helper cells and mounting cytotoxic T lymphocytes (CTL) against tumor antigens [13, 14]. It was reported that alterations of HLA class II molecules are relevant to the development of cancer and metastatic progression. Individuals who inherit specific alleles of the highly polymorphic HLA class II (DP, DQ, or DRB) genes have increased risk of cancer, cervix [5] and breast [6, 15], due to alterations in the immune surveillance. However, their role in PCa is still unclear.

HLA-DPA1 rs3077 SNP is located in the 3′ untranslated region (3′ UTR) of gene. Thus, it has no role on the structure and function of HLA-DPA1 protein. However, this SNP may affect the transcription, processing, stability, or translation of HLA-DPA1 mRNA, and it may affect the transcription, processing, stability, or translation of HLA-DPA1 mRNA [16].

The current study aimed to appraise the relevance of HLA-DPA1 rs3077 (A/G) SNP to PCa risk and/or severity. Results clarified that HLA-DPA1 rs3077 GG genotype was more frequent among PCa patients and the associated OR was 2.546 (0.964–6.726), indicating that persons carrying this genotype are at 2.5 higher risk to develop PCa. This was confirmed on the allele level as the frequency of G allele was higher among PCa patients than the control group while A allele frequency was significantly decreased among PCa patients (*P*=0.034^*∗*^) (protective allele). Correlation analysis between PSA ratio and different genotypes in the PCa group did not show any significant association between this SNP and severity of PCa (*P*=0.856). In agreement with this study, Jiang et al. [5] have genotyped HLA-DP (rs3077 and rs9277535) and HLA-DQ (rs2856718 and rs7453920) SNP and found consistent associations between HLA-DP rs3077 and cervical cancer risks. They reported HLA-DP rs3077 as a candidate susceptibility marker for cervical cancer in Chinese females.

These results are in accordance with many researchers who found statistically significant association between cancer risk and HLA-DP rs3077 polymorphisms [5, 6, 17–19]. HLA-DP rs3077 and rs9277535 were reported as susceptibility markers for cervical cancer among Chinese females [5]. Ghazy et al. [6] have found significant association between HLA-DP1 rs3077 AA genotype and breast cancer risk. Zhang et al. [17] have investigated the association between HLA-DP rs3077 and susceptibility to hepatocellular carcinoma (HCC) among HBV infected patients. They noticed significant association of rs3077 with HCC susceptibility in the Asian population. Jia et al. [18] have assumed that human leukocyte antigens (HLA) could be susceptibility alleles that might contribute to cervical cancer among females infected by human papillomavirus (HPV). They found that HLA-DP rs3077 SNP is markedly associated with cervical cancer risk (OR = 1.37, 95% CI = 1.04–1.80). Cao et al. [19] have investigated the association between HLA-DP/DQ SNP and acute myeloid leukemia (AML) risk. They have genotyped HLA-DP (rs3077 G > A and rs9277535 G > A) and HLA-DQ (rs2856718 A > G and rs7453920 G > A) in a case-control study using real-time PCR. They found significant associations between increased AML risk and HLA-DP (rs3077 and rs9277535) SNP. They suggested HLA-DP and HLA-DQ loci as susceptibility alleles for AML in Han Chinese.

On the other hand, some researchers found a significant lack of association between breast cancer and HLA-DQA1 in southern Taiwanese women [4]. Liao et al. [20] have performed meta-analysis to appraise the association between HLA-DP/DQ SNP and HCC development. However, no association was found with HCC development (dominant model, rs3077, OR = 0.86, 95% CI = 0.62–1.18). Cheng et al. have reported that HLA-DP rs3077 SNP were not significantly associated with cervical cancer risk in Chinese population [21].

These discrepancies between results might be attributed to the PCa heterogeneity around the World [12], location of the SNP [22] or ethnicity of the studied population, as ethnicity is closely linked to hereditary factors in the genesis of PCa [9, 12]. What limits our study is the small sample size, which is attributed to low disease burden in our locality.

## 5. Conclusion

HLA-DPA1 rs3077 G alleles could be a risk factor for development of PCa while A allele is protective. However, HLA-DPA1 rs3077 SNP has no relation to PCa severity. Further validation studies on larger scale of different ethnic groups with analysis of the biological function are warranted.

## Figures and Tables

**Table 1:** Patients' demographics.

	Prostate cancer (*n* = 40)	Control (*n* = 40)	*t* or *U*	*P*
Age (years)	59.9 ± 9.2	56.3 ± 10.3	*t* = 1.635	0.106
BMI	22.68 ± 6.17	24.68 ± 4.17		0.105
Smoking	13 (8%)	8 (3.2%)		0.12
*Medical comorbidity*				
Diabetes mellitus	1 (2.5%)	0 (0%)		
Hypertension	3 (7.5%)	4 (10.0%)		0.107
Hepatic fibrosis	1 (2.5%)	0 (0%)		
Nephropathy	2 (5.0%)	3 (7.5%)		
S. urea (mg/dl)	26.6 ± 5.7	14.3 ± 2.8	t = 12.123^*∗*^	<0.001^*∗*^
S. creatinine (mg/dl)	1.22 ± 0.23	0.85 ± 0.04	t = 9.598^*∗*^	<0.001^*∗*^
Total serum PSA (ng/ml)	1.8 ± 0.97	1 ± 0.83	U = 370.5^*∗*^	<0.001^*∗*^
Free serum PSA (ng/ml)	0.51 ± 0.32	0.6 ± 0.73	U = 670.0	0.227
*PSA ratio (free-to-total PSA) (%)*				
Mean ± SD	37 ± 6.2	18.5 ± 3.6	U = 0.0^*∗*^	<0.001^*∗*^
Median (min–max)	35.6 (27.3–56.59)	17.32 (10.65–25)		

*t*, student's *t*-test; *U,* Mann–Whitney test; *P*, *P* value for comparing between the studied groups. ^*∗*^Statistically significant at *P* ≤ 0.05.

**Table 2:** Comparison between the two studied groups according to different genotypes.

	Cancer		Control®		*χ* ^2^ (p)	*P*_1_	OR (95% CI)
	Observed	Expected	Observed	Expected			
HLA-DP rs077A	(*n* = 40)		(*n* = 40)				
GG	17 (42.5%)	19.6	9 (22.5%)	11.6	6.031^*∗*^ (^MC^p = 0.049^*∗*^)	0.059	2.546 (0.964–6.726)
AG	22 (55%)	16.8	25 (62.5%)	19.9		0.496	0.733 (0.300–1.791)
AA	1 (2.5%)	3.6	6 (15%)	8.6		0.081	0.145 (0.017–1.268)
HWE	0.050		0.104				
Allele frequency	(*n* = 80)		(*n* = 80)				
G	56 (70%)		43 (53.8%)		4.478^*∗*^ (0.034^*∗*^)	0.035^*∗*^	2.008 (1.049–3.844)
A	24 (30%)		37 (46.3%)			0.035^*∗*^	0.498 (0.260–0.954)

OR, odds ratio; CI, confidence interval; LL, lower limit; UL, upper limit; ®, reference group; *χ*^2^, chi-square test; MC, Monte Carlo; HWE, *P* value for Hardy-Weinberg. If *P* < 0.05, not consistent with HWE. *P*, *P* value for comparing between the studied groups; *P*_1_, *P* value for odds ratio (univariate regression analysis). ^*∗*^Statistically significant at *P* ≤ 0.05.

**Table 3:** Relation between PSA ratio and different genotypes in PCa group (*n* = 40).

HLA-DPA1 rs3077 A	*N*	PSA ratio (%)			Test of sig.	*P*
		Min–Max	Mean ± SD	Median		
GG	17	28.8–56.6	37.2 ± 7.1	35	*U* = 180.0	0.856
AG	22	27.2–57.5	37.1 ± 7.9	37.2		
AA	1^#^	30^#^				

*U,* Mann–Whitney test; *H,* Kruskal–Wallis test. *P*, *P* value for association between different categories. ^#^Excluded from the association due to small number of cases (*n* = 1).

## Data Availability

All data are available with the corresponding author.
